# Smokers are less likely than non-smokers to seek help for a lung cancer ‘alarm’ symptom

**DOI:** 10.1136/thoraxjnl-2015-208063

**Published:** 2016-02-24

**Authors:** C Friedemann Smith, K L Whitaker, K Winstanley, J Wardle

**Affiliations:** 1 Department of Epidemiology and Public Health, Health Behaviour Research Centre, University College London, London, UK; 2 School of Health Sciences, University of Surrey, Guildford, Surrey, UK

**Keywords:** Lung Cancer, Psychology, Tobacco and the lung

## Abstract

**Background:**

The majority (>85%) of lung cancer cases are linked with smoking, and prognosis is poor because it is often diagnosed at a late stage. One contributor to late-stage diagnosis could be patient delay in help-seeking. We investigated the help-seeking behaviour of smokers and non-smokers for a recent lung cancer alarm symptom.

**Methods:**

A health survey was sent to 4913 men and women aged >50 years through through General Practice. It included questions on symptoms experienced in the past 3 months (from a checklist), help-seeking (Yes/No) for each symptom and demographic characteristics including smoking status. Univariable and multivariable binary logistic regression analyses were used to assess the association between smoking status and help-seeking for a cough or hoarseness.

**Results:**

Among 2042 participants (42% response rate), 280 (14%) reported ‘cough or hoarseness’ in the past 3 months; of whom 22% were current smokers. Being a smoker was associated with reduced likelihood of help-seeking (OR 0.44; 95% CI 0.23 to 0.83), even after adjusting for demographic factors (OR 0.46; 95% CI 0.21 to 1.00).

**Conclusions:**

Delay in help-seeking in smokers for a symptom that is potentially indicative of lung cancer is a cause for concern. Future research could usefully address the psychological mechanisms through which help-seeking in smokers is hindered.

## Background

Smoking is strongly linked to the development of lung cancer, playing a causal role in >85% of cases and prognosis is poor with <10% 5-year survival.^1^ A possible contributor is the time taken for patients to be diagnosed, which is reportedly longer than other common cancers.^2^ The majority of cancers are diagnosed when patients seek medical advice for a symptom,^3^ and the time taken for an individual to notice a new symptom, appraise it as worthy of medical attention, and seek help from their doctor, have been called the appraisal and help-seeking intervals.^4^ Understanding these intervals is vital in efforts to promote earlier cancer diagnosis because influences on help-seeking are complex and behaviour is driven by multiple factors. In smokers, drivers of help-seeking may be even more complex because their habit adds additional barriers; evidence shows that smokers fail to seek help when they experience ‘alarm’ symptoms for lung cancer.^5^ However, much of this research is retrospective or speculative, and both these methods could influence previous findings.

This report describes a ‘health survey’ study to establish whether smokers reporting cough or hoarseness in the past 3 months are less likely to have sought help than non-smokers.

## Methods

We mailed 4913 adults aged ≥50 years in England a ‘health survey’ through four English General Practices. Responders were asked whether they had experienced any of 14 cancer alarm symptoms in the past 3 months (including persistent cough or hoarseness) taken from the Cancer Awareness Measure and Be Clear on Cancer campaigns,^6^ ^7^ whether they smoked (yes, occasionally, previously, never), and whether they had sought help from their General Practitioner (GP) for reported symptoms (see online supplementary material for Health Survey). The present analysis focuses specifically on behaviour among those reporting ‘persistent cough or hoarseness’. Univariable and multivariable binary logistic regressions assessed the association between smoking status and help-seeking. Analyses were carried out using SPSS V.22.0.

10.1136/thoraxjnl-2015-208063.supp1 Supplementary data

## Results

A total of 2042 (42%) surveys were returned. Among participants, 280 (14%) reported ‘persistent cough or hoarseness’ in the past 3 months, of whom 60 (22%) were smokers. Smokers were more likely to be male, aged ≥60 years, married or cohabiting and have not attended university (data not shown). Just under half of smokers (n=129; 46%) had sought help for their symptom from their GP (table 1). Univariable logistic regression (table 2) showed that being a smoker was associated with a lower likelihood of help-seeking (OR 0.44; 95% CI 0.23 to 0.83): 55% of non-smokers had sought help compared with 35% of smokers. In multivariable logistic regression analyses the effect of smoking status remained significant when all demographic factors were controlled (OR 0.46; 95% CI 0.21 to 1.00). Smoking status was not associated with help-seeking for the other 13 ‘alarm’ symptoms (p>0.05).

**Table 1:** Demographic characteristics and help-seeking in whole sample and sample reporting cough or hoarseness

Demographic characteristic (N)	Subgroups	Whole sample (N=2042)*, N (%)	Sample reporting cough or hoarseness (N=280)*, N (%)	Help-seeking in sample reporting cough or hoarseness (% of those in subcategory)
Smoking status†	Non-smokers	1777 (88%)	219 (78%)	111 (55%)
	Smokers	252 (12%)	60 (22%)	18 (35%)
Sex	Men	936 (46%)	105 (38%)	41 (43%)
	Women	1085 (54%)	172 (62%)	87 (55%)
Age	Under 60 years	622 (34%)	84 (34%)	39 (51%)
	60 years and older	1194 (66%)	162 (66%)	80 (53%)
Ethnicity	Non-white	1919 (95%)	14 (6%)	10 (77%)
	White	99 (5%)	216 (94%)	118 (49%)
Marital status	Not married/cohabiting	649 (32%)	121 (43%)	53 (49%)
	Married/ cohabiting	1372 (68%)	158 (57%)	77 (52%)
Education	Below university	1259 (63%)	187 (68%)	91 (53%)
	University	740 (37%)	86 (32%)	34 (44%)
Employment	Not working	1194 (59%)	185 (66%)	95 (57%)
	Working	822 (41%)	93 (34%)	34 (40%)

*Numbers may not add up to the total N of each sample because of missing data.

†Those who never or previously smoked were classified as non-smokers, those who currently or occasionally smoked were classified as smokers.

**Table 2:** Tests of help-seeking in smokers and non-smokers for persistent cough or hoarseness

	Subgroups (N, %)	Univariable ORs for help-seeking for persistent cough (unadjusted), 95% CI	Multivariable ORs for help-seeking for persistent cough (adjusted), 95% CI
Smoking status	Never/ ex-smokers	1.00	1.00
	Current smokers	0.44 (0.23 to 0.83)	0.46 (0.21 to 1.00)
Sex	Men	1.00	1.00
	Women	1.61 (0.97 to 2.70)	1.95 (1.07 to 3.54)
Age	Under 60 years	1.00	1.00
	60 years and older	1.07 (0.62 to 1.86)	0.57 (0.28 to 1.16)
Ethnicity	White	1.00	1.00
	Non-white	3.42 (0.92 to 12.73)	3.43 (0.86 to 13.63)
Marital status	Not married/cohabiting	1.00	1.00
	Married/ cohabiting	1.13 (0.69 to 1.85)	1.24 (0.68 to 2.26)
Education	Below university	1.00	1.00
	University	0.69 (0.40 to 1.18)	0.66 (0.35 to 1.23)
Employment	Not working	1.00	1.00
	Working	0.50 (0.30 to 0.85)	0.35 (0.17 to 0.71)

Tests for multicollinearity were run. All tolerance statistics were above 0.76 and all variance inflation factor statistics were below 1.31.

## Discussion

In this study 14% of participants had experienced a persistent cough or hoarseness in the past 3 months, of which a fifth were smokers. Smoking status was significantly and independently associated with help-seeking, with smokers less likely to seek help than non-smokers. These findings support previous studies where smokers have avoided medical advice for lung cancer symptoms.^5^ ^8^

Participants were presented with a ‘health survey’ rather than a ‘cancer survey’. This was done to simulate the circumstances in which new symptoms appear in real life, and avoid participants over-reporting help-seeking as a consequence of being alerted to cancer. It is thus likely that the responses are a good reflection of usual behaviour. We also asked participants about the past 3 months, making recall bias or failings of memory less likely.

A limitation is that we focused on one lung cancer alarm symptom combination (persistent cough or hoarseness). Despite being established symptoms of lung cancer, these are also common symptoms of other benign diseases. In contrast, haemoptysis is comparatively rare and more specific and tends to promote help-seeking.^9^ So we may not have captured how participants would react when faced with more ‘alarming’ symptoms. However, cough and hoarseness are among the most common early symptoms of lung cancer,^9^ and so these findings are potentially important. Another limitation is that we did not collect data on whether participants sought help for previous episodes of cough or hoarseness prior to the 3-month window. Research has shown that a previous ‘all clear’ diagnosis can delay help-seeking for persistent or new symptoms.^10^ Renzi *et al*'s review did not include papers examining the effect of a non-cancer diagnosis on symptoms of lung cancer and so this is a potential area for further investigation.

Another area for future research could identify and test strategies which aim to improve help-seeking by targeting mechanisms contributing to delay. Normalising symptoms, whereby an individual interprets a negative change in health as part of normal bodily functions, is a key cause of delay in help-seeking in patients with lung cancer.^5^ Normalising symptoms provides a context in which our findings can be interpreted, a cough or hoarseness is simply a normal result of smoking that is no cause for concern, and an area for awareness campaigns to target. For example, campaigns could put time limits on symptoms, for example, ‘visit your GP if you have a cough that lasts for more than three weeks’, and placing this information in areas targeting smokers, for example on cigarette packaging. If this was combined with messages encouraging help-seeking from GPs, other mechanisms by which help-seeking in smokers is hindered, such as the stigma around smoking, could also be addressed.

## Conclusion

Much effort has been put into establishing characteristics that make someone more or less likely to seek help for symptoms that could be indicative of cancer. This study has shown that in a community sample, smokers are less likely than non-smokers to seek help for a common symptom of lung cancer, despite being at higher risk of cancer. Future research addressing potential mechanisms contributing to delay in help-seeking in smokers could identify strategies through which help-seeking might be encouraged.
