# Supplementary material for: Smokers are less likely than non-smokers to seek help for a lung cancer ‘alarm’ symptom
Source: Thorax. 2016 Feb 24;71(7):659–61. doi: 10.1136/thoraxjnl-2015-208063 (PMC4941149; doi:10.1136/thoraxjnl-2015-208063)
Supplement: Supplementary data [file thoraxjnl-2015-208063supp.pdf]

Participant number:

HEALTH SURVEY

Thank you for agreeing to complete this survey.

- It should take no longer than 20 minutes to complete. You may, however, take as long as you wish.
- Your answers will be sent directly to the research team at University College London (UCL) and will be treated in strict confidence. No individual responses will be seen by the General Practitioner (GP).
- Please return the survey in the freepost envelope (no stamp required). By returning the survey you are consenting to take part in the study.

If you have any difficulties completing this survey or have any questions about it, please call **020 7679 1735**

Thank you

FIRST, SOME QUESTIONS ABOUT YOUR VIEWS ON GOING TO SEE THE GP

The following statements are things some people say about visiting the GP. Next to EACH STATEMENT, please tick the box that best reflects your level of agreement.

The GP will NOT be made aware of your individual responses. Your answers will be sent directly to the UCL research team.

	Strongly disagree	Slightly disagree	Neither agree or disagree	Slightly agree	Strongly agree
I would feel confident discussing any type of symptom with the GP	☐	☐	☐	☐	☐
I feel embarrassed talking to the GP about my symptoms	☐	☐	☐	☐	☐
Visiting the GP with symptoms that may not be serious is a waste of my time	☐	☐	☐	☐	☐
It is difficult to make an appointment with the GP	☐	☐	☐	☐	☐
If I thought a symptom might be serious, I would be too scared to see the GP	☐	☐	☐	☐	☐
I spend time "scanning" my body for changes.	☐	☐	☐	☐	☐
I have lots of things (other than my health) to worry about	☐	☐	☐	☐	☐
It is difficult for me to arrange transport to the GP's surgery	☐	☐	☐	☐	☐
It is a waste of the GP's time to see patients with symptoms that may not be serious	☐	☐	☐	☐	☐
I am too busy to make time to visit the GP	☐	☐	☐	☐	☐
The GP gives me the opportunity to ask questions	☐	☐	☐	☐	☐
I am very sensitive to changes in my body.	☐	☐	☐	☐	☐
The GP encourages me to mention all my health concerns during the consultation	☐	☐	☐	☐	☐
The GP is difficult to talk to	☐	☐	☐	☐	☐
I worry about what the GP might find	☐	☐	☐	☐	☐
I pay close attention to changes in my body	☐	☐	☐	☐	☐

Approximately how many times have you been to see the GP in the last 3 months?

Have not been	Once	Twice	Three or more times
☐	☐	☐	☐

Generally speaking, do you think that most people your age visit the GP…

Less often than they should	About as often as they should	More often than they should
☐	☐	☐

Compared with other people your age, do you think that you visit the GP…

Less often than most people	About the same as most people	More often than most people
☐	☐	☐

THESE QUESTIONS ARE ABOUT YOUR EXPERIENCE OF SYMPTOMS IN THE **LAST 3 MONTHS**.

Please note: If you experience symptoms persistently (i.e. they don't go away), you should go to your GP for advice.

In the last 3 months, have you had the following….? *Please answer yes or no for each symptom*	Approximately when did the symptom start/when did you first notice it? (Please give your best guess)	How long did the symptom last?	Have you been concerned the symptom might be serious?	How much has the symptom interfered with your life?	What do you think may have caused the symptom? (Please list as many things as come to mind)	Approximately how long after the symptom began did you contact the GP about it? (If you are unsure, please give your best guess)
**Unexplained weight loss**<br>☐ **YES** ⟶<br>☐ **NO**	☐ Less than 1 week ago<br>☐ Less than 2 weeks ago<br>☐ Less than 1 month ago<br>☐ Less than 6 weeks ago<br>☐ Less than 3 months ago<br>☐ 3 months ago or longer	Number of days:<br>________days<br>Or:<br>☐ On-going	☐ Not at all<br>☐ A little bit<br>☐ Moderately<br>☐ Quite a bit<br>☐ Extremely	☐ Not at all<br>☐ A little bit<br>☐ Moderately<br>☐ Quite a bit<br>☐ Extremely	________<br>________<br>________<br>________	☐ Did not contact the GP<br>☐ Not contacted the GP yet, but plan to<br>☐ Within 1-2 weeks of noticing symptom<br>☐ Within 1 month of noticing symptom<br>☐ Within 6 weeks of noticing symptom<br>☐ Within 3 months of noticing symptom<br>☐ After more than 3 months of noticing symptom
**Unexplained lump**<br>☐ **YES** ⟶<br>☐ **NO**	☐ Less than 1 week ago<br>☐ Less than 2 weeks ago<br>☐ Less than 1 month ago<br>☐ Less than 6 weeks ago<br>☐ Less than 3 months ago<br>☐ 3 months ago or longer	Number of days:<br>________days<br>Or:<br>☐ On-going	☐ Not at all<br>☐ A little bit<br>☐ Moderately<br>☐ Quite a bit<br>☐ Extremely	☐ Not at all<br>☐ A little bit<br>☐ Moderately<br>☐ Quite a bit<br>☐ Extremely	________<br>________<br>________<br>________	☐ Did not contact the GP<br>☐ Not contacted the GP yet, but plan to<br>☐ Within 1-2 weeks of noticing symptom<br>☐ Within 1 month of noticing symptom<br>☐ Within 6 weeks of noticing symptom<br>☐ Within 3 months of noticing symptom<br>☐ After more than 3 months of noticing symptom
**Change in the appearance of a mole or a new mole**<br>☐ **YES** ⟶<br>☐ **NO**	☐ Less than 1 week ago<br>☐ Less than 2 weeks ago<br>☐ Less than 1 month ago<br>☐ Less than 6 weeks ago<br>☐ Less than 3 months ago<br>☐ 3 months ago or longer	Number of days:<br>________days<br>Or:<br>☐ On-going	☐ Not at all<br>☐ A little bit<br>☐ Moderately<br>☐ Quite a bit<br>☐ Extremely	☐ Not at all<br>☐ A little bit<br>☐ Moderately<br>☐ Quite a bit<br>☐ Extremely	________<br>________<br>________<br>________	☐ Did not contact the GP<br>☐ Not contacted the GP yet, but plan to<br>☐ Within 1-2 weeks of noticing symptom<br>☐ Within 1 month of noticing symptom<br>☐ Within 6 weeks of noticing symptom<br>☐ Within 3 months of noticing symptom<br>☐ After more than 3 months of noticing symptom
**Persistent change in bowel habits**<br>(persistent means doesn't go away)<br>☐ **YES** ⟶<br>☐ **NO**	☐ Less than 1 week ago<br>☐ Less than 2 weeks ago<br>☐ Less than 1 month ago<br>☐ Less than 6 weeks ago<br>☐ Less than 3 months ago<br>☐ 3 months ago or longer	Number of days:<br>________days<br>Or:<br>☐ On-going	☐ Not at all<br>☐ A little bit<br>☐ Moderately<br>☐ Quite a bit<br>☐ Extremely	☐ Not at all<br>☐ A little bit<br>☐ Moderately<br>☐ Quite a bit<br>☐ Extremely	________<br>________<br>________<br>________	☐ Did not contact the GP<br>☐ Not contacted the GP yet, but plan to<br>☐ Within 1-2 weeks of noticing symptom<br>☐ Within 1 month of noticing symptom<br>☐ Within 6 weeks of noticing symptom<br>☐ Within 3 months of noticing symptom<br>☐ After more than 3 months of noticing symptom

In the last 3 months, have you had the following....?<br>**Please answer for each symptom**	Approximately when did the symptom start/when did you first notice it?<br>(Please give your best guess)	How long did the symptom last?	Have you been concerned the symptom might be serious?	How much has the symptom interfered with your life?	What do <u>you</u> think may have caused the symptom?<br>(Please list as many things as come to mind)	Approximately how long after the symptom began did you contact the GP about it?<br>(If you are unsure, please give your best guess)
**Blood in urine**<br>☐ **YES** ⟶<br>☐ **NO**	☐ Less than 1 week ago<br>☐ Less than 2 weeks ago<br>☐ Less than 1 month ago<br>☐ Less than 6 weeks ago<br>☐ Less than 3 months ago<br>☐ 3 months ago or longer	Number of days:<br>_________days<br>Or:<br>☐ On-going	☐ Not at all<br>☐ A little bit<br>☐ Moderately<br>☐ Quite a bit<br>☐ Extremely	☐ Not at all<br>☐ A little bit<br>☐ Moderately<br>☐ Quite a bit<br>☐ Extremely		☐ Did not contact the GP<br>☐ Not contacted the GP yet, but plan to<br>☐ Within 1-2 weeks of noticing symptom<br>☐ Within 1 month of noticing symptom<br>☐ Within 6 weeks of noticing symptom<br>☐ Within 3 months of noticing symptom<br>☐ After more than 3 months of noticing symptom
**Persistent change in bladder habits**<br>☐ **YES** ⟶<br>☐ **NO**	☐ Less than 1 week ago<br>☐ Less than 2 weeks ago<br>☐ Less than 1 month ago<br>☐ Less than 6 weeks ago<br>☐ Less than 3 months ago<br>☐ 3 months ago or longer	Number of days:<br>_________days<br>Or:<br>☐ On-going	☐ Not at all<br>☐ A little bit<br>☐ Moderately<br>☐ Quite a bit<br>☐ Extremely	☐ Not at all<br>☐ A little bit<br>☐ Moderately<br>☐ Quite a bit<br>☐ Extremely		☐ Did not contact the GP<br>☐ Not contacted the GP yet, but plan to<br>☐ Within 1-2 weeks of noticing symptom<br>☐ Within 1 month of noticing symptom<br>☐ Within 6 weeks of noticing symptom<br>☐ Within 3 months of noticing symptom<br>☐ After more than 3 months of noticing symptom
**Any breast changes**<br>☐ **YES** ⟶<br>☐ **NO**	☐ Less than 1 week ago<br>☐ Less than 2 weeks ago<br>☐ Less than 1 month ago<br>☐ Less than 6 weeks ago<br>☐ Less than 3 months ago<br>☐ 3 months ago or longer	Number of days:<br>_________days<br>Or:<br>☐ On-going	☐ Not at all<br>☐ A little bit<br>☐ Moderately<br>☐ Quite a bit<br>☐ Extremely	☐ Not at all<br>☐ A little bit<br>☐ Moderately<br>☐ Quite a bit<br>☐ Extremely		☐ Did not contact the GP<br>☐ Not contacted the GP yet, but plan to<br>☐ Within 1-2 weeks of noticing symptom<br>☐ Within 1 month of noticing symptom<br>☐ Within 6 weeks of noticing symptom<br>☐ Within 3 months of noticing symptom<br>☐ After more than 3 months of noticing symptom
**Persistent unexplained pain**<br>☐ **YES** ⟶<br>☐ **NO**	☐ Less than 1 week ago<br>☐ Less than 2 weeks ago<br>☐ Less than 1 month ago<br>☐ Less than 6 weeks ago<br>☐ Less than 3 months ago<br>☐ 3 months ago or longer	Number of days:<br>_________days<br>Or:<br>☐ On-going	☐ Not at all<br>☐ A little bit<br>☐ Moderately<br>☐ Quite a bit<br>☐ Extremely	☐ Not at all<br>☐ A little bit<br>☐ Moderately<br>☐ Quite a bit<br>☐ Extremely		☐ Did not contact the GP<br>☐ Not contacted the GP yet, but plan to<br>☐ Within 1-2 weeks of noticing symptom<br>☐ Within 1 month of noticing symptom<br>☐ Within 6 weeks of noticing symptom<br>☐ Within 3 months of noticing symptom<br>☐ After more than 3 months of noticing symptom
**Persistent difficulty swallowing**<br>☐ **YES** ⟶<br>☐ **NO**	☐ Less than 1 week ago<br>☐ Less than 2 weeks ago<br>☐ Less than 1 month ago<br>☐ Less than 6 weeks ago<br>☐ Less than 3 months ago<br>☐ 3 months ago or longer	Number of days:<br>_________days<br>Or:<br>☐ On-going	☐ Not at all<br>☐ A little bit<br>☐ Moderately<br>☐ Quite a bit<br>☐ Extremely	☐ Not at all<br>☐ A little bit<br>☐ Moderately<br>☐ Quite a bit<br>☐ Extremely		☐ Did not contact the GP<br>☐ Not contacted the GP yet, but plan to<br>☐ Within 1-2 weeks of noticing symptom<br>☐ Within 1 month of noticing symptom<br>☐ Within 6 weeks of noticing symptom<br>☐ Within 3 months of noticing symptom<br>☐ After more than 3 months of noticing symptom

In the last 3 months, have you had the following….? *Please answer for each symptom*	Approximately when did the symptom start/when did you first notice it? (Please give your best guess)	How long did the symptom last?	Have you been concerned the symptom might be serious?	How much has the symptom interfered with your life?	What do you think may have caused the symptom? (Please list as many things as come to mind)	Approximately how long after the symptom began did you contact the GP about it? (If you are unsure, please give your best guess)
**Persistent cough or hoarseness**<br>☐ **YES** ⟶<br>☐ **NO**	☐ Less than 1 week ago<br>☐ Less than 2 weeks ago<br>☐ Less than 1 month ago<br>☐ Less than 6 weeks ago<br>☐ Less than 3 months ago<br>☐ 3 months ago or longer	Number of days:<br>________days<br>Or:<br>☐ On-going	☐ Not at all<br>☐ A little bit<br>☐ Moderately<br>☐ Quite a bit<br>☐ Extremely	☐ Not at all<br>☐ A little bit<br>☐ Moderately<br>☐ Quite a bit<br>☐ Extremely	________<br>________<br>________<br>________	☐ Did not contact the GP<br>☐ Not contacted the GP yet, but plan to<br>☐ Within 1-2 weeks of noticing symptom<br>☐ Within 1 month of noticing symptom<br>☐ Within 6 weeks of noticing symptom<br>☐ Within 3 months of noticing symptom<br>☐ After more than 3 months of noticing symptom
**Rectal bleeding (i.e. bleeding from the back passage or blood in the bowel motions)**<br>☐ **YES** ⟶<br>☐ **NO**	☐ Less than 1 week ago<br>☐ Less than 2 weeks ago<br>☐ Less than 1 month ago<br>☐ Less than 6 weeks ago<br>☐ Less than 3 months ago<br>☐ 3 months ago or longer	Number of days:<br>________days<br>Or:<br>☐ On-going	☐ Not at all<br>☐ A little bit<br>☐ Moderately<br>☐ Quite a bit<br>☐ Extremely	☐ Not at all<br>☐ A little bit<br>☐ Moderately<br>☐ Quite a bit<br>☐ Extremely	________<br>________<br>________<br>________	☐ Did not contact the GP<br>☐ Not contacted the GP yet, but plan to<br>☐ Within 1-2 weeks of noticing symptom<br>☐ Within 1 month of noticing symptom<br>☐ Within 6 weeks of noticing symptom<br>☐ Within 3 months of noticing symptom<br>☐ After more than 3 months of noticing symptom
**Other unexplained bleeding**<br>☐ **YES** ⟶<br>☐ **NO**	☐ Less than 1 week ago<br>☐ Less than 2 weeks ago<br>☐ Less than 1 month ago<br>☐ Less than 6 weeks ago<br>☐ Less than 3 months ago<br>☐ 3 months ago or longer	Number of days:<br>________days<br>Or:<br>☐ On-going	☐ Not at all<br>☐ A little bit<br>☐ Moderately<br>☐ Quite a bit<br>☐ Extremely	☐ Not at all<br>☐ A little bit<br>☐ Moderately<br>☐ Quite a bit<br>☐ Extremely	________<br>________<br>________<br>________	☐ Did not contact the GP<br>☐ Not contacted the GP yet, but plan to<br>☐ Within 1-2 weeks of noticing symptom<br>☐ Within 1 month of noticing symptom<br>☐ Within 6 weeks of noticing symptom<br>☐ Within 3 months of noticing symptom<br>☐ After more than 3 months of noticing symptom
**Abdominal bloating (i.e. bloating of your tummy or belly)**<br>☐ **YES** ⟶<br>☐ **NO**	☐ Less than 1 week ago<br>☐ Less than 2 weeks ago<br>☐ Less than 1 month ago<br>☐ Less than 6 weeks ago<br>☐ Less than 3 months ago<br>☐ 3 months ago or longer	Number of days:<br>________days<br>Or:<br>☐ On-going	☐ Not at all<br>☐ A little bit<br>☐ Moderately<br>☐ Quite a bit<br>☐ Extremely	☐ Not at all<br>☐ A little bit<br>☐ Moderately<br>☐ Quite a bit<br>☐ Extremely	________<br>________<br>________<br>________	☐ Did not contact the GP<br>☐ Not contacted the GP yet, but plan to<br>☐ Within 1-2 weeks of noticing symptom<br>☐ Within 1 month of noticing symptom<br>☐ Within 6 weeks of noticing symptom<br>☐ Within 3 months of noticing symptom<br>☐ After more than 3 months of noticing symptom
**A sore that does not heal**<br>☐ **YES** ⟶<br>☐ **NO**	☐ Less than 1 week ago<br>☐ Less than 2 weeks ago<br>☐ Less than 1 month ago<br>☐ Less than 6 weeks ago<br>☐ Less than 3 months ago<br>☐ 3 months ago or longer	Number of days:<br>________days<br>Or:<br>☐ On-going	☐ Not at all<br>☐ A little bit<br>☐ Moderately<br>☐ Quite a bit<br>☐ Extremely	☐ Not at all<br>☐ A little bit<br>☐ Moderately<br>☐ Quite a bit<br>☐ Extremely	________<br>________<br>________<br>________	☐ Did not contact the GP<br>☐ Not contacted the GP yet, but plan to<br>☐ Within 1-2 weeks of noticing symptom<br>☐ Within 1 month of noticing symptom<br>☐ Within 6 weeks of noticing symptom<br>☐ Within 3 months of noticing symptom<br>☐ After more than 3 months of noticing symptom

PLEASE NOTE: IF YOU HAVE SYMPTOMS PERSISTENTLY, YOU SHOULD GO TO YOUR GP FOR ADVICE

THE NEXT FEW QUESTIONS ARE ABOUT YOUR GENERAL HEALTH

Compared with other people your age, would you say your health is…?

Poor	Fair	Good	Very good	Excellent
☐	☐	☐	☐	☐

Do you have any illnesses or conditions that affect your daily life?

Yes	No
☐	☐

Do you have a diagnosis of any of the following conditions/illnesses? ****Please tick all that apply***

Arthritis	☐	Cholesterol problems	☐	High blood pressure	☐
Cancer	☐	Depression	☐	Kidney problems	☐
Circulation problems	☐	Diabetes	☐	Stroke	☐
Chest problems	☐	Heart problems	☐	Other (please specify)	☐ ____________________

Have you had any of the following investigations/tests in the LAST TWO YEARS? (excluding investigations performed after injuries) ****Please tick all that apply***

Ultrasound scan	☐	Colonoscopy/Sigmoidoscopy	☐
CT or MRI scan	☐	Biopsy	☐
Endoscopy	☐	PSA test	☐
Mammogram	☐	Other test (please specify)	☐ ________________________
Chest X-Ray	☐	Have not had any investigations/tests	☐

If you have, approximately how many months ago was your most recent investigation/test?

_____________ months ago

If more than once, please refer to the LAST time you had any of the above tests

On a day-to-day basis, how much do you worry about …

	Not at all	A little	Moderately	Quite a bit	A lot
Heart Disease	☐	☐	☐	☐	☐
Cancer	☐	☐	☐	☐	☐
Alzheimer's Disease	☐	☐	☐	☐	☐

THE NEXT QUESTIONS ARE ABOUT YOUR KNOWLEDGE OF SOME ILLNESSES

In the table below are a number of symptoms. For each symptom, please tick as many illnesses as you think might apply.

	Heart disease	Cancer	Asthma	Don't know
Chest pain	☐	☐	☐	☐
Unexplained lump	☐	☐	☐	☐
Unexplained weight loss	☐	☐	☐	☐
Persistent change in bowel habits	☐	☐	☐	☐
Blood in urine	☐	☐	☐	☐
Persistent change in bladder habits	☐	☐	☐	☐
Shortness of breath	☐	☐	☐	☐
Persistent unexplained pain	☐	☐	☐	☐
Persistent difficulty swallowing	☐	☐	☐	☐
Change in the appearance of a mole or a new mole	☐	☐	☐	☐
Rectal bleeding (i.e. bleeding from the back passage or blood in the bowel motions)	☐	☐	☐	☐
Other unexplained bleeding	☐	☐	☐	☐
Any breast changes	☐	☐	☐	☐
Abdominal bloating (i.e. bloating of your tummy or belly)	☐	☐	☐	☐
Feeling your heart pound or race	☐	☐	☐	☐
Persistent cough or hoarseness	☐	☐	☐	☐
A sore that does not heal	☐	☐	☐	☐

How much attention do you pay to information about health or medical topics on TV, radio, magazines or newspapers?

A lot	Some	A little	None at all	I avoid them
☐	☐	☐	☐	☐

	Very difficult	Fairly difficult	Fairly easy	Very easy	Don't know
How easy do you find it to understand what the GP says to you?	☐	☐	☐	☐	☐
How easy do you find it to understand the leaflets that come with medication?	☐	☐	☐	☐	☐
How easy do you find it to follow the instructions on medication?	☐	☐	☐	☐	☐

At the moment, the NHS has screening programmes for breast and cervical cancer for women, and bowel cancer for men and women. Have you ever participated in any of the following programmes? If yes, please state how many times you have participated in each.

Name of programme	Have you participated in this programme?	If yes, how many times?
Bowel cancer screening (sometimes known as the faecal occult blood test, FOB test or stool kit test)	Yes ☐<br>No ☐<br>Not sure ☐	Once ☐<br>2-3 times ☐<br>4 times or more ☐<br>Not sure ☐
Breast cancer screening (sometimes known as mammography)	Yes ☐<br>No ☐<br>Not sure ☐	Once ☐<br>2-3 times ☐<br>4 times or more ☐<br>Not sure ☐
Cervical cancer screening (sometimes known as PAP smear)	Yes ☐<br>No ☐<br>Not sure ☐	Once ☐<br>2-3 times ☐<br>4 times or more ☐<br>Not sure ☐

FINALLY, A FEW QUESTIONS ABOUT YOU TO HELP US ANALYSE THE RESULTS OF THE SURVEY

Please enter today's date: d d | m m | y y y y

**Are you:**	Male ☐ Female ☐
**What is your date of birth?**	d d / m m / y y y y

What is your ethnic group?

White British ☐	Indian ☐	Black Caribbean ☐	Chinese ☐	White & Black African ☐
White Irish ☐	Pakistani ☐	Black African ☐	White & Black Caribbean ☐	Prefer not to say ☐
Other White background ☐	Bangladeshi ☐	White Asian ☐	Other (please specify) ☐ …………………………………………………………	

What is the highest level of education qualification you have obtained?

Degree or higher degree	☐	O Level/GCSE ('Standard grades' in Scotland)	☐
Higher education qualification below degree level	☐	No formal qualifications	☐
A-levels ('Highers' in Scotland)	☐	Other (please specify)	☐
ONC/BTEC	☐	________________________________	

What is your marital status?

Single/never married	Married/living with partner	Civil partnership	Divorced/Separated	Widowed
☐	☐	☐	☐	☐

Does your household own a car or van?

No	Yes, one	Yes, two or more
☐	☐	☐

**Approximately how many days a week do you drink alcohol?** (If unknown, please give your best guess)	Every day	Most days	1-2 days a week	Less than once a week
	☐	☐	☐	☐

What are your current living arrangements?

Home owned outright	Home owned with mortgage	Rent from Local Authority/Housing Association	Rent privately	Other (e.g. living with family /friends)
☐	☐	☐	☐	☐

What is your postcode?* ☐☐☐☐☐☐☐

**This information will not be used to contact you and will not be shared with any third parties. It is for research purposes only.*

What is your current employment status?

Employed full-time	☐	Full-time homemaker	☐
Employed part-time	☐	Retired	☐
Unemployed	☐	Studying	☐
Self-employed	☐	Disabled or too ill to work	☐

How tall are you?
(If unknown, please give your best guess) ________________ cm OR ________feet___________inches

How much do you weigh?
(If unknown, please give your best guess) ________________Kg OR _________stone__________pounds

Do you smoke? (please tick the ONE box below that best describes you)

Yes, I am a current smoker	Yes, I smoke occasionally	Not now, but I used to smoke	No, I have never smoked
☐	☐	☐	☐

Have any friends or family members that are close to you ever been diagnosed with the following illnesses?
**Please tick the box next to all that apply*

	Parent/brother/ sister/child	Other family member	Close friend	No/not applicable
Heart disease	☐	☐	☐	☐
Cancer	☐	☐	☐	☐
Asthma	☐	☐	☐	☐

FURTHER INFORMATION

In approximately three months, we are planning to contact a few people to tell us more about their experiences. Would you be prepared to be contacted again? Yes ☐ No ☐

If yes, please provide your details below (this information will be treated in the strictest confidence and will be kept separately from your questionnaire):

Name ______________________________________

Address ____________________________________

____

____

____

Email ______________________________________

Telephone ___________________________________

If you would like to receive a summary of the results from this survey when it is completed, please fill in your contact details below.

Information provided here will not be used to contact you about taking part in research.

Name ______________________________________

Address ____________________________________

____

____

____

Email ______________________________________

IMPORTANT:

IF YOU ARE WORRIED ABOUT ANY SYMPTOMS OR ARE EXPERIENCING PERSISTENT SYMPTOMS, GO TO THE GP FOR ADVICE

Thank you for completing this survey.
